# Risk Analysis Index for Estimation of 30-Day Postoperative Mortality in Hip Fractures

**DOI:** 10.1001/jamanetworkopen.2025.12689

**Published:** 2025-05-29

**Authors:** Nithin K. Gupta, Hikmat R. Chmait, Vikram Gill, Morgan Turnow, Taylor Manes, Benjamin C. Taylor, Jack W. Weick, Christian Bowers

**Affiliations:** 1 Campbell University School of Osteopathic Medicine, Lillington, North Carolina; 2 REAM Orthopedics, Columbus, Ohio; 3 Bowers Neurosurgical Frailty and Outcomes Data Science Lab, Flint, Michigan; 4 Larner College of Medicine at The University of Vermont, Burlington; 5 Mayo Clinic Alix School of Medicine, Phoenix, Arizona; 6 Department of Orthopaedic Surgery, OhioHealth Grant Medical Center, Columbus, Ohio; 7 Hurley Neurological Center, Hurley Medical Center, Flint, Michigan

## Abstract

**Importance:**

Hip fractures present a substantial public health challenge, with projections of more than 500 000 per year by 2040. As such, frailty indices such as the Revised Risk Analysis Index (RAI) and the Modified Five-Item Frailty Index (mFI-5) have been recently investigated as metrics for preoperative risk stratification for these patients.

**Objective:**

To understand the accuracy of frailty, as measured by the RAI and the mFI-5, for estimating 30-day mortality following surgically managed hip fractures.

**Design, Setting, and Participants:**

This retrospective cross-sectional analysis used the American College of Surgeons’ National Surgical Quality Improvement database. Patients aged 65 years old with surgically managed traumatic hip fracture from 2015 to 2019 were included. Frailty was evaluated using the RAI, a 5-domain scale with 14 weighted variables graded from 0 to 81, and the mFI-5, a 2-domain scale with 5 unweighted variables graded from 0 to 5; for both, a higher score denotes worse frailty. Data collection occurred from May to June 2024.

**Exposure:**

Diagnosis of hip fracture and undergoing surgical fixation, hemiarthroplasty, or total hip arthroplasty.

**Main Outcomes and Measures:**

The primary outcome was 30-day mortality. Multivariable regression was conducted to assess the estimating value of frailty scales. Discriminatory accuracy was assessed using a receiver operating characteristic curve and quantified using a C-statistic.

**Results:**

The cohort consisted of 114 359 patients (70 038 female [69.9%]; median [IQR] age, 84 [77-89] years) with 51 071 prefrail patients (44.7%) according to the mFI-5 and 31 883 very frail patients (27.9%) according to the RAI comprising the largest frailty groups. Increasing frailty status was associated with greater odds ratio (OR) for 30-day mortality for both the mFI-5 (prefrail OR, 1.35 [95% CI, 1.24-1.47]; frail OR, 2.11 [95% CI, 1.94-2.30]; severely frail OR, 3.53 [95% CI, 3.20-3.90]; *P* < .001 for all) and RAI (normal OR, 1.55 [95% CI, 1.35-1.79]; frail OR, 2.97 [95% CI, 2.59-3.42]; very frail OR, 6.17 [95% CI, 5.38-7.08]; *P* < .001 for all). The RAI demonstrated superior discriminatory accuracy for 30-day mortality compared with the mFI-5 (area under the receiver operating characteristic curve, 0.73 [95% CI, 0.72-0.73] vs 0.61 [95% CI, 0.60-0.62]; *P* < .001).

**Conclusions and Relevance:**

In this cross-sectional study of 114 359 patients, the RAI demonstrated superior odds and discriminatory accuracy for estimating 30-day mortality following surgical management of hip fractures. The RAI may be considered as a risk stratification tool for orthopedic surgeons to adjunct surgical planning, thereby reducing postoperative mortality.

## Key Points

**Question:**

Does the Risk Analysis Index (RAI), as a frailty assessment tool, demonstrate superior discrimination compared with the Modified Five-Item Frailty Index (mFI-5) in estimating mortality among patients with surgically stabilized hip fracture?

**Findings:**

In this cross-sectional study of 114 359 patients from the American College of Surgeons National Surgical Quality Improvement Program, the RAI demonstrated superior discrimination compared with the mFI-5 for estimating mortality within 30 days following surgical fixation of hip fractures.

**Meaning:**

These findings suggest that the RAI may be used as a preoperative risk stratification tool to adjunct surgical decision-making for patients with hip fracture, thereby reducing mortality.

## Teaser

This cross-sectional study examines the accuracy of frailty, as measured by the Risk Analysis Index and the Modified Five-Item Frailty Index, for estimating 30-day mortality following surgically managed hip fractures.

## Introduction

Hip fractures represent a major public health challenge, with a lifetime prevalence in nearly 20% of women and 10% of men.^1^ The increasing incidence and substantial burden hip fractures impose on individuals and health care systems worldwide illustrate the need to properly evaluate and manage this patient population. The estimated projection of annual new hip fractures by 2050 ranges from 500 000 to 1 000 000, with an estimated annual cost of approximately $10.3 billion to $15.2 billion.^2,3^ The growing prevalence of these fractures is largely associated with an aging population, which, coupled with susceptibility to falls, escalates the number of such incidents annually.^4^ Beyond the immediate medical needs, hip fractures exert substantial social and financial strain on hospitals because of prolonged hospital stays, extensive rehabilitation requirements, and the high risk of complications including mortality.^5^ With hip fracture mortality reaching nearly 25% and permanent disability rates spanning from 32% to 80% of all patients, a clear need to maximize outcomes is evident.^6,7^ The overarching objective of managing hip fractures is to restore function and mobility, minimize length of hospital stay, and reduce health care costs. To successfully achieve these objectives, timely and definitive operative management of hip fractures is critical. This not only enhances functional outcomes but mitigates the economic impacts on the health care system.^8^ Given the complexities associated with these patients, who often present with varying degrees of frailty and comorbid conditions, preoperative assessments are indispensable.^9^

Frailty, which is distinct from multimorbidity, refers to a multidimensional state characterized by decreased physiological reserve, leading to increased vulnerability to stressors.^10^ Although it is related, multimorbidity is defined as having 2 or more diseases or the accumulation of multiple chronic diseases.^11^ Thus, a physiologic frail state may stem from multimorbidity or predispose an individual to the development of chronic diseases. Importantly, frailty has been identified as a crucial factor influencing postoperative outcomes in elderly patients.^12,13,14^ Therefore, incorporating a robust frailty assessment into preoperative planning is paramount for optimizing resource allocation, improving patient outcomes, and refining surgical strategies. In the orthopedic surgery literature, the Modified Five-Item Frailty Index (mFI-5) is the most common frailty index and has been extensively used to estimate postoperative outcomes.^15,16,17,18^ Although well established, the mFI-5 has been criticized as a measure of multimorbidity.^19,20^ As such there has been a growing body of evidence supporting the Risk Analysis Index (RAI), which incorporates multiple domains of frailty and has been shown to have greater discriminatory accuracy.^13,21,22,23^

The present study seeks to compare RAI and mFI-5 in terms of their accuracy in estimating 30-day postoperative outcomes in patients undergoing operative stabilization of hip fractures. By determining which index demonstrates superior discrimination, we aim to contribute to the refinement of preoperative evaluation practices, thereby enhancing the surgical management and prognostication of this vulnerable patient population.

## Methods

### Data Source

The American College of Surgeons National Surgical Quality Improvement Program (ACS-NSQIP) is a national variable-based database consisting of reports from approximately 700 hospitals nationwide.^23^ This database has been validated previously in estimating outcomes using frailty.^24,25^ The data used in this study were retrieved from the 2015 to 2020 ACS-NSQIP participant use data files. Data collection occurred from May to June 2024. Data elements included patient demographics, clinical characteristics, and postoperative outcomes. Race and ethnicity are considered 2 unique variables in the ACS-NSQIP database (starting in 2008) and are collected by a certified Surgical Clinical Reviewer through various methods, including medical record review.^26^ Race was categorized by the ACS-NSQIP as American Indian or Alaska Native, Asian, Black or African American, Native Hawaiian or Pacific Islander, White, unknown, or not reported, and race combinations with low frequency (patients with multiple race options selected and some other race). Ethnicity was categorized as Hispanic or not Hispanic.^27^ Race and ethnicity were included in this study to understand the demographic composition of the study cohort and because they have been shown to play a role in outcomes for hip fracture patients.^28^ Because the ACS-NSQIP is a publicly accessible database containing deidentified patient data, this study is exempt from institutional review board approval and/or patient approval; thus, neither was sought. This study was performed by the Strengthening the Reporting of Observational Studies in Epidemiology (STROBE) reporting guidelines, with clear identification of study objective, methods, participant selection (eFigure in Supplement 1), discussion of key results, limitations, and generalizability.^29^

### Patient Selection

Patients aged 65 years or older with a diagnosis of hip fracture who underwent surgical fixation, hemiarthroplasty, or total hip arthroplasty were included in the study. Diagnosis codes were defined by the *International Classification of Diseases, Ninth Revision* and *International Statistical Classification of Diseases and Related Health Problems, Tenth Revision* (eTable 1 in Supplement 1). Surgical management codes were defined using *Current Procedural Terminology *codes (eTable 1 in Supplement 1).

### Modified Five-Item Frailty Index

The mFI-5 is a frailty index originally developed by Chimukangara et al^24^ and validated using the NSQIP. It consists of the 5 clinical variables reported within the NSQIP (4 unique comorbidity variables and 1 variable for independent function), each with a score of 1 to a maximum score of 5 (eTable 2 in Supplement 1). The following mFI-5 frailty cutoffs were used: 0, nonfrail; 1, prefrail; 2, frail; and 3 or higher, severely frail. This score has been extensively used in the orthopedic literature, including traumatic hip fractures.^15,16^

### Risk Analysis Index

The RAI was originally described by Hall et al^19^ as a measure of frailty and subsequently was validated using surgical patients identified in the Veterans Affairs Surgical Quality Improvement Program. The RAI was later recalibrated using the NSQIP to validate it for use in nonveteran surgical patients.^25^ The RAI was created as a more accurate assessment of the frailty phenotype, encompassing 5 frailty domains: physical (comorbidity), functional, social, nutritional, and cognitive. It is calculated using 11 weighted variables, with a score range of 0 to 81 (eTables 3 and 4 in Supplement 1). Tiered groups were created on the basis of the recently developed RAI User Guide,^30^ which defined 4 clinically relevant groups: robust, normal, frail, and very frail. The RAI cutoff scores were defined according to mortality rates in reference to the normal group (robust, 0.5 times normal; frail, 2 times normal; and very frail, 4 times normal). On the basis of this method, the RAI score cutoffs were calculated as follows: robust, 20 to 25; normal, 26 to 28; frail, 29 to 32; and very frail, 33 or higher.^30^ These groups correspond to the nonfrail, prefrail, frail, and severely frail groups of the mFI-5, respectively.

### Population Characteristics and Preoperative Variables

Baseline population characteristics were recorded and included age, sex, and race. Descriptive comorbidities included were those used in the mFI-5 and RAI frailty score calculations (diabetes, chronic obstructive pulmonary disease, hypertension, congestive heart failure, functional status, cancer diagnosis, unintentional weight loss, kidney failure or dialysis, poor appetite, shortness of breath at rest, and residency other than home).

### Outcomes and Complications

The primary outcome of this study was the 30-day mortality rate. Secondary outcomes included discharge disposition, unplanned readmission, unplanned reoperation, extended length of stay (eLOS; ie, >1 day), and major complications within 30 days. Major complications consisted of the Clavien-Dindo IV complications.^31^

### Statistical Analysis

Statistical analysis was performed using R Studio software version 4.4.1 (R Project for Statistical Computing). Baseline demographic variables that were continuous were reported as median with IQR. Preoperative comorbidities and 30-day outcome variables were reported as incidence. Multivariable analysis was performed controlling for age, sex, race, transfer status, total operation time, days from admission to operation, and surgical procedure. The effect sizes of RAI and mFI-5 on 30-day outcomes were reported as odds ratio (OR) and 95% CI. Discriminatory accuracy of each frailty model was assessed using receiver operating characteristic (ROC) analysis with area under the ROC curve (AUROC) and/or C-statistic used to quantify. The discriminatory accuracy of the frailty models was compared using the DeLong test. Significance was indicated by 2-sided *P *< .05.

## Results

### Study Population Characteristics

The study cohort consisted of 114 359 patients (median [IQR] age, 84 [77-89] years) of whom 70 038 (69.9%) were female (Table). There were 95 733 patients (87.3%) with American Society of Anesthesiologists classification 3 or higher, and the most common comorbidities were hypertension (77 891 patients [68.1%]) and diabetes (20 555 patients [17.9%]). The most common surgical procedure was fixation of trochanteric fractures with intramedullary implant (48 243 patients [42.2%]).

**Table.** Demographic and Clinical Characteristics of Patients With Hip Fracture by mFI-5 and RAI Frailty Status

Characteristic	Patients, No. (%) (N = 114 359)							
	mFI-5				RAI			
	Nonfrail (n = 23 111 [20.2%])	Prefrail (n = 51 071 [44.7%])	Frail (n = 31 430 (27.5%])	Severely frail (n = 8747 [7.6%])	Robust (n = 25 572 [22.4%])	Normal (n = 27 217 [23.7%])	Frail (n = 29 687 [26.0%])	Very frail (n = 31 883 [27.9%])
Age, median (IQR), y	82 (74-88)	85 (78-90)	84 (77-89)	83 (77-79)	72 (69-76)	83 (80-86)	88 (83-90)	89 (83-90)
Sex								
Male	6637 (28.7)	14 703 (28.8)	9829 (31.3)	3252 (37.2)	5322 (20.8)	4700 (17.3)	11 057 (37.2)	13 342 (41.8)
Female	16 474 (71.3)	36 368 (71.2)	21 601 (68.7)	5495 (62.8)	20 250 (79.2)	22 517 (82.7)	18 630 (62.8)	18 630 (58.4)
Race and ethnicity								
American Indian or Alaska Native	84 (0.4)	162 (0.3)	120 (0.4)	40 (0.5)	124 (0.5)	105 (0.4)	96 (0.3)	81 (0.3)
Asian	500 (2.2)	1122 (2.2)	849 (2.7)	239 (2.7)	562 (2.2)	688 (2.5)	632 (2.1)	828 (2.6)
Black	436 (1.9)	1477 (2.9)	1194 (3.8)	398 (4.6)	899 (3.5)	751 (2.8)	790 (2.7)	1065 (3.3)
Pacific Islander	17 (0.1)	44 (0.1)	51 (0.2)	14 (0.2)	27 (0.1)	26 (0.1)	34 (0.1)	39 (0.1)
White	15 082 (65.3)	36 099 (70.7)	21 404 (68.1)	5825 (66.6)	18 354 (71.8)	19 482 (71.6)	20 649 (69.6)	20 645 (64.8)
Unknown	6271 (27.1)	12 164 (23.8)	7810 (24.8)	2231 (25.5)	560 (2.2)	6163 (22.6)	7485 (25.2)	9224 (28.9)
American Society of Anesthesiologists classification, median (IQR)	3 (2-3)	3 (3-3)	3 (3-4)	3 (3-4)	3 (2-3)	3 (3-3)	3 (3-3)	3 (3-4)
Time to operation, median (IQR), d	1 (0-1)	1 (1-1)	1 (1-1)	1 (1-2)	1 (0-1)	1 (1-1)	1 (1-1)	1 (1-2)
Preoperative comorbidities								
Diabetes	0	2095 (4.1)	12 563 (40.0)	5897 (67.4)	5636 (22.0)	4990 (18.3)	4801 (16.2)	5128 (16.1)
Chronic obstructive pulmonary disease	0	2186 (4.3)	5873 (18.7)	4173 (47.7)	2959 (11.6)	2617 (9.6)	2873 (9.7)	3783 (11.9)
Hypertension	0	39 808 (77.9)	29 498 (93.9)	8585 (98.1)	15 566 (60.9)	19 139 (70.3)	21 108 (71.1)	22 078 (69.2)
Congestive heart failure	0	310 (0.6)	1660 (5.3)	2410 (27.6)	52 (0.2)	182 (0.7)	760 (2.6)	3386 (10.6)
Independent functional status	23 111 (100.0)	44 399 (86.9)	18 164 (57.8)	2351 (26.9)	25 572 (100.0)	26 811 (98.5)	26 828 (90.4)	8814 (27.6)
Cancer diagnosis	416 (1.8)	750 (1.5)	458 (1.5)	172 (2.0)	0	0	0	1796 (5.6)
Unintentional weight loss	370 (1.6)	945 (1.9)	779 (2.5)	277 (3.2)	0	70 (0.3)	293 (1.0)	2008 (6.3)
Kidney failure or dialysis	376 (1.6)	1856 (3.6)	2376 (7.6)	1248 (14.3)	0	152 (0.6)	592 (2.0)	5112 (16.0)
Poor appetite	1480 (6.4)	3780 (7.4)	3116 (9.9)	1108 (12.7)	0	280 (1.0)	1172 (3.9)	8032 (25.2)
Shortness of breath at rest	35 (0.2)	256 (0.5)	461 (1.5)	405 (4.6)	83 (0.3)	129 (0.5)	254 (0.9)	691 (2.2)
Admitted from home	4741 (20.5)	12 314 (24.1)	9825 (31.3)	3397 (38.8)	4088 (16.0)	3994 (14.7)	9320 (31.4)	12 875 (40.4)
Operative technique								
Total hip arthroplasty	1769 (7.7)	2674 (5.2)	1163 (3.7)	263 (3.0)	3040 (11.9)	1287 (4.7)	883 (3.0)	659 (2.1)
Hemiarthroplasty	2983 (12.9)	6806 (13.3)	4177 (13.3)	1203 (13.8)	3027 (11.8)	3689 (13.6)	4029 (13.6)	4424 (13.9)
Open treatment (fixation or prosthetic)	7069 (30.6)	15 998 (31.3)	9540 (30.4)	2575 (29.4)	7296 (28.5)	8328 (30.6)	9290 (31.3)	10 268 (32.2)
Plate or screw fixation (sliding compression screw)	2057 (8.9)	4271 (8.4)	2761 (8.8)	807 (9.2)	2120 (8.3)	2386 (8.8)	2524 (8.5)	2866 (9.0)
Intramedullary implant (cephalomedullary nail)	9233 (40.0)	21 322 (41.7)	13 789 (43.9)	3899 (44.6)	10 089 (39.5)	11 527 (42.4)	12 961 (43.7)	13 666 (42.9)
Outcomes								
Length of stay, median (IQR), d	5 (3-7)	5 (4-7)	5 (4-8)	6 (4-9)	5 (3-7)	5 (4-7)	5 (4-8)	6 (4-8)
Nonhome discharge	16 278 (70.4)	38 520 (75.4)	23 451 (74.6)	6352 (72.6)	16 820 (65.8)	21 560 (79.2)	23 626 (79.6)	22 595 (70.9)
Major complications	948 (4.1)	2964 (5.8)	2374 (7.6)	1022 (11.7)	1066 (4.2)	1498 (5.5)	2068 (7.0)	2676 (8.4)
Unplanned readmission	1318 (5.7)	3969 (7.8)	2997 (9.5)	1167 (13.3)	1592 (6.2)	2035 (7.5)	2578 (8.7)	3246 (10.2)
Unplanned reoperation	480 (2.1)	1203 (2.4)	788 (2.5)	264 (3.0)	618 (2.4)	665 (2.4)	675 (2.3)	779 (2.4)
Death	727 (3.1)	2454 (4.8)	2345 (7.5)	1060 (12.1)	344 (1.3)	702 (2.6)	1736 (5.8)	3804 (11.9)

Abbreviations: mFI-5, Modified Five-Item Frailty Index; RAI, Risk Analysis Index.

The median (IQR) RAI score was 29 (26-33), and the median (IQR) mFI-5 score was 1 (1-2). According to the RAI, 25 572 patients (22.4%) were classified as robust, 27 217 (23.7%) as normal, 29 687 (26.0%) as frail, and 31 883 (27.9%) as very frail. For the mFI-5, 23 111 patients (20.2%) were classified as nonfrail, 51 071 (44.7%) as prefrail, 31 430 (27.5%) as frail, and 8747 (7.6%) as severely frail. There were 5913 patients (19.9%) classified as frail by the RAI and the mFI-5 (eTable 5 in Supplement 1).

### Primary End Point: Frailty and 30-Day Mortality

**Figure 1.**
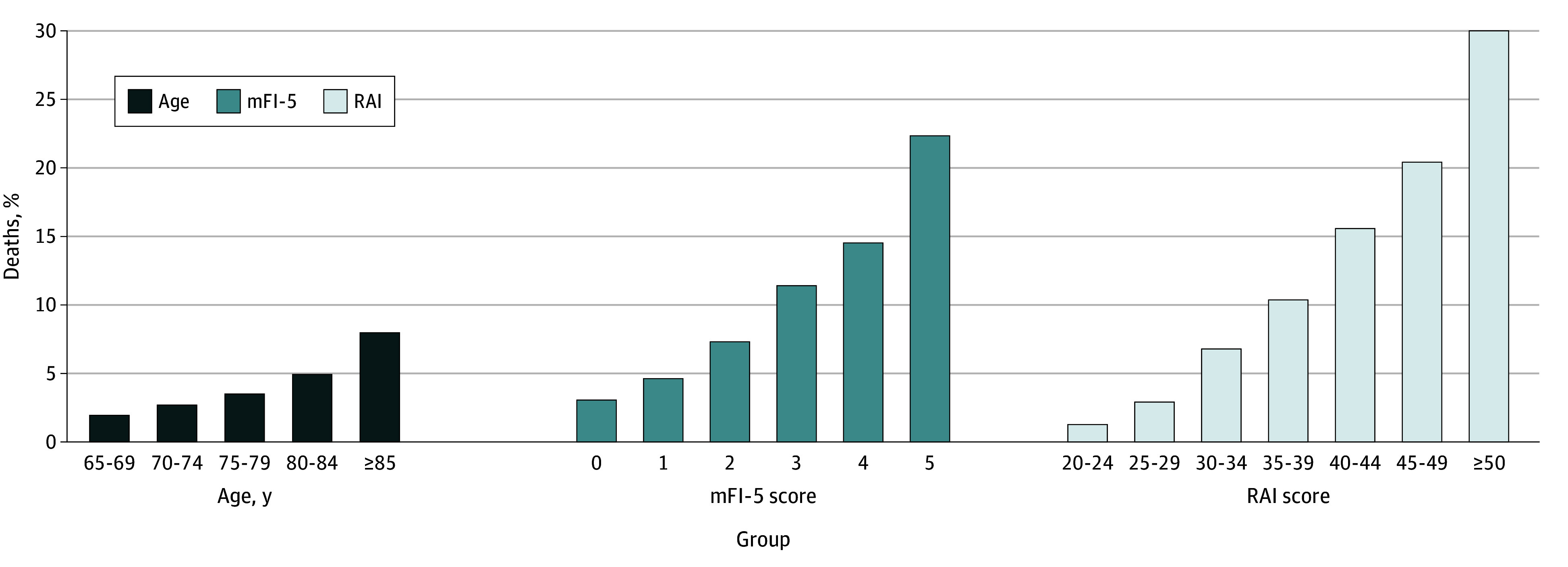
Postoperative Mortality at 30 Days by Age, Modified Five-Item Frailty Index (mFI-5) Status, and Risk Analysis Index (RAI) Tier Bar chart shows the percentage of patients who died within 30 days after surgical management for hip fracture by age, mFI-5 status, and RAI tier.

There were 6586 deaths (5.8%) overall within 30 days. The percentage mortality by mFI-5 score ranged from 727 deaths (3.15%), for a score of 0, to 22 deaths (23.4%), for a score of 5. For RAI, percentage mortality ranged from 254 deaths (1.2%), for a score of 20 to 24, to 105 deaths (30.7%), for a score of 50 or higher (Figure 1).

**Figure 2.**
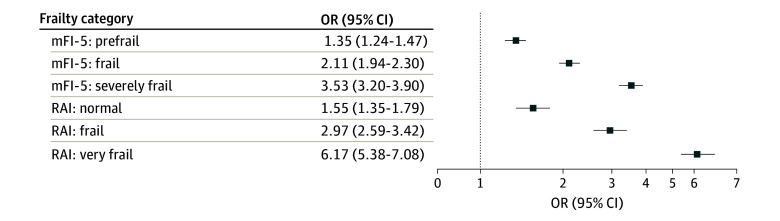
Multivariable Regression for Odds of Mortality Estimated by Frailty Plot shows results of multivariable analysis for odds of mortality within 30 days estimated by mortality for Modified Five-Item Frailty Index (mFI-5) (reference, nonfrail) and Risk Analysis Index (RAI) (reference, robust). Logistic regression controls for confounding variables, including age, sex, race, operative time, time from admission to surgery, surgery type, and transfer status. OR indicates odds ratio.

Multivariable regression controlling for age, sex, race, operative time, time to surgery, surgical technique, and transfer status was performed to evaluate the estimating value of RAI and mFI-5 (Figure 2). Increasing frailty was associated with greater odds of mortality using the mFI-5 (prefrail OR, 1.35 [95% CI, 1.24-1.47]; frail OR, 2.11 [95% CI, 1.94-2.30]; severely frail OR, 3.53 [95% CI, 3.20-3.90]; *P* < .001 for all) and RAI (normal OR, 1.55 [95% CI, 1.35-1.79]; frail OR, 2.97 [95% CI, 2.59-3.42]; very frail OR, 6.17 [95% CI, 5.38-7.08]; *P* < .001 for all).

**Figure 3.**
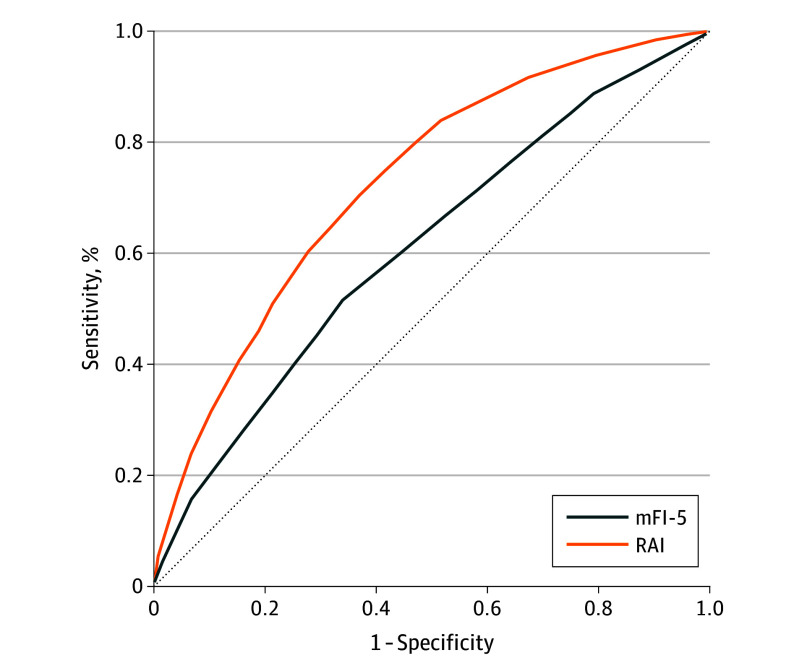
Area Under the Receiver Operating Characteristic Curve (AUROC) Analysis for Discriminatory Accuracy of Frailty Graph shows discriminatory accuracy of the Risk Analysis Index (RAI) and Modified Five-Item Frailty Index (mFI-5) for 30-day mortality using AUROC analysis. The C statistic for RAI was significantly greater than that for mFI-5 (AUROC, 0.73 [95% CI, 0.72-0.73] vs 0.61 [95% CI, 0.60-0.62]; *P* < .001).

AUROC analysis was used to compare the discriminatory accuracies of the mFI-5 and RAI for 30-day mortality (Figure 2). The RAI demonstrated superior discriminatory accuracy compared with the mFI-5 for the primary end point of 30-day mortality (AUROC, 0.73 [95% CI, 0.72-0.73] vs 0.61 [95% CI, 0.60-0.62]; *P* < .001) (Figure 3 and eTable 6 in Supplement 1).

### Secondary End Points

Multivariable analysis was also conducted to understand the estimating value of frailty on the secondary end points eLOS, nonhome discharge (NHD), readmission, and reoperation. Increasing frailty as measured by the RAI and mFI-5 had increased odds for all secondary end points except NHD (eTable 7 in Supplement 1). Discriminatory accuracy for all secondary end points for both the RAI and mFI-5 demonstrated C-statistics less than or equal to 0.60 (eTable 6 in Supplement 1).

## Discussion

In this cross-sectional study of a large cohort of 114 359 patients with hip fracture who were operatively stabilized, worsening frailty as measured by the RAI was significantly associated with mortality and eLOS within 30 days of injury. Furthermore, the RAI had greater discriminatory accuracy compared with the mFI-5 for estimating mortality, eLOS, and NHD. This study is one of the first to report the use of RAI in orthopedics and demonstrates the association of frailty with adverse outcomes in surgically managed hip fractures. Furthermore, these results suggest that RAI may be more useful than the mFI-5 as a preoperative risk assessment tool to estimate mortality following hip fracture surgery.

Recently, frailty has gained rapid interest within orthopedic surgery as a tool for preoperative risk stratification and surgical candidate selection. Although there remains no consensus within the literature for a criterion standard frailty scale in orthopedics, the mFI and its derivatives remain the most commonly used.^15^ In our multivariable analysis, the mFI-5 was significantly associated with postoperative mortality following hip fractures, similar to previous reports utilizing the mFI-5, 11-Factor Modified Frailty Index (mFI-11), and Age-Adjusted Modified Frailty Index (aamFI).^31,32,33,34^ These findings were strengthened by a retrospective study of 316 patients, showing an association of frailty as measured by the Comprehensive Geriatric Assessment–based Frailty Index with 2-year mortality following hip fractures.^35^

Interestingly, in our study, the RAI was shown to have greater odds and superior discrimination for estimating mortality compared with the mFI-5. These findings are crucial and may suggest that the RAI is a more accurate measure of frailty compared with the mFI-5. Fundamentally, the mFI-5 is based on the accumulation of deficits model, which has been criticized as a measure of multimorbidity and primarily validated retrospectively.^24,25^ Conversely, the RAI is a prospectively validated index, using weighted measures from 5 domains of frailty that provide a more-comprehensive representation of the accumulation of deficits model on which it, too, is based.^25^ Unlike the mFI-5, the RAI accounts for age and sex, which are independent from the phenotypic definition of frailty but are key elements of the frailty state defined by physiologic deficit accumulation.^36,37^ The well-established concept of age-related cellular and/or molecular damage and the body of evidence supporting that deficit accumulation are more lethal for men compared with women are examples of the crucial multidimensional representation of the frailty state by the RAI.^37^

These conceptual advantages are demonstrated in the literature as the RAI has been shown extensively to outperform the mFI-5 and other commonly used frailty measures, such as the HFRS, for estimating postoperative outcomes, especially in the neurosurgical literature.^38,39^ Investigations of discriminatory accuracy of other frailty measures in patients with hip fracture reported in the literature, such as the Clinical Frailty Index, mFI-5, mFI-11, Modified 19-Item Frailty Index, and aaMFI, tend to report lower AUROC values, further strengthening the RAI’s role in estimating mortality in this cohort.^33,40,41^ Another advantage of the RAI lies in the granular nature of its scoring calculation. As the median age of our cohort was 84 years, it allows frailty tiers to be adjusted on the basis of the demographic of the pathology, allowing us to use a score of 29 or higher for frail, thereby accounting for an overall baseline frail cohort (unlike the mFI-5).

In general, both the mFI-5 and RAI were associated with eLOS, NHD, Clavien-Dindo IV complications, readmission, and reoperation via multivariable analysis. A previous meta-analysis^42^ of 22 studies found that increasing frailty in patients with hip fractures was significantly associated with increased eLOS, abnormal discharge, and perioperative complications, further supporting our findings. Also, frailty scales such as the Chinese-Canadian Study of Health and Aging Clinical Frailty Scale and the mFI are reported to be associated with readmission and revision procedures.^43,44^ Given the relatively large body of evidence supporting frailty’s role in nonfatal adverse outcomes, our AUROC analysis demonstrated poor (C-statistic <0.60) discriminatory accuracy for both RAI and mFI-5.^45^ This may demonstrate the impact of social, environmental, and/or clinical factors outside of frailty. For example, literature has shown that NHD following hip fractures can be influenced by insurance status, patient preference, marital status, race, or even hospital discharge guidelines.^46,47,48,49^ Given these results, frailty does not provide adequate risk stratification for nonfatal adverse outcomes, which is important to consider when interpreting the utility of frailty in hip fractures. However, with the risk of mortality increasing 3 to 4 times within 1 year of hip fractures, the RAI is an essential tool to help reduce the estimated 36% 1-year mortality rate.^50,51^

The application of frailty tools clinically has been challenging, and there is a movement away from further identification of a criterion standard toward supporting the use of any frailty tool.^52^ This is, in part, owing to studies demonstrating similar estimating value and risk discrimination between various frailty models.^53^ However, it is important to note that the RAI, unlike many other tools in the literature, has been extensively validated for frailty in surgical patients. Furthermore, it has been used effectively at the bedside to screen large volumes of patients (in as little as 30 seconds). As a multidimensional tool with actionable variables, the RAI can identify patients with hip fracture who are at greater risk for mortality and allow for targeted interventions and/or resource allocation, such as support for improved activities of daily living or appetite stimulation.^30^ As the RAI is intended for use as an adjunct, it may be a helpful starting point for goals of care discussions with patients and their families, which has been shown to be an underutilized yet crucial part of the treatment pathway in patients with hip fracture.^54,55,56^

### Limitations

The limitations of the current study are largely attributed to those that are inherent to studies utilizing the ACS-NSQIP database. Our results demonstrated strong risk discrimination for 30-day mortality by the RAI; however, risk calibration was not possible given the dataset. Although the RAI has been previously calibrated for use in surgical patients, future studies may seek to perform risk calibration analysis specifically in patients with hip fracture.^25^ In addition, there was a small subset of patients who were excluded because of incomplete data, which precluded calculation of the RAI frailty score.

The generalizability of our study may be limited because our cohort was primarily composed of White women; however, this may be a reflection of the demographic profile of hip fractures.^57,58^ In addition, although the mFI-5 is the most commonly used frailty tool in the orthopedic literature, prospective studies are required to compare the RAI’s clinical feasibility with frailty tools currently used by orthopedic surgeons.^59,60^ Although these limitations exist, this study demonstrates that the previously validated RAI is able to both capture the multidimensional aspects of frailty, while providing superior estimating capability for the adverse outcomes most associated with frailty. Thus, the utilization of RAI should be considered for estimating 30-day mortality rates in older patients.

## Conclusions

In this cross-sectional study of a large multicenter national database, frailty, as measured by the RAI, was demonstrated to be independently associated with 30-day mortality and nonfatal adverse outcomes following surgical fixation of hip fractures. In addition, the RAI had superior discriminatory accuracy compared with the mFI-5 for mortality. To our knowledge, this is the first study using a large cohort to demonstrate the utility of the RAI over the mFI-5 in patients with hip fracture, especially for estimating mortality. The results of this study have high clinical relevance and suggest the RAI should be used over the mFI-5 for preoperative risk stratification before operative stabilization of hip fractures.

## Notes

Supplement 1. **eTable 1.** *ICD* and *CPT* codes used for patient selection **eTable 2.** mFI-5 scoring criteria **eTable 3.** RAI Scoring criteria **eTable 4.** Weighting of age variable by presence of cancer for RAI **eFigure.** CONSORT Flow Diagram **eTable 5.** Crosstabulation of mFI-5 and RAI **eTable 6.** Discriminatory accuracy of the RAI and mFI-5 for primary outcome and secondary outcomes **eTable 7. **Multivariable analysis controlling for age, sex, race, operative time, and transfer status, for prediction of 30-day outcomes by the RAI and mFI-5

Supplement 2.
Data Sharing Statement
