# Supplementary material for: Risk Analysis Index for Estimation of 30-Day Postoperative Mortality in Hip Fractures
Source: JAMA Netw Open. 2025 May 29;8(5):e2512689. doi: 10.1001/jamanetworkopen.2025.12689 (PMC12123473; doi:10.1001/jamanetworkopen.2025.12689)
Supplement: Supplement 1. — eTable 1. ICD and CPT codes used for patient selection eTable 2. mFI-5 scoring criteria eTable 3. RAI Scoring criteria eTable 4. Weighting of age variable by presence of cancer for RAI eFigure. CONSORT Flow Diagram eTable 5. Crosstabulation of mFI-5 and RAI eTable 6. Discriminatory accuracy of the RAI and mFI-5 for primary outcome and secondary outcomes eTable 7. Multivariable analysis controlling for age, sex, race, operative time, and transfer status, for prediction of 30-day outcomes by the RAI and mFI-5 [file jamanetwopen-e2512689-s001.pdf]

Supplemental Online Content

Gupta N, Chmait HR, Gill V, et al. Risk analysis index for estimation of 30-day postoperative mortality in hip fractures. *JAMA Netw Open*. 2025;8(5):e2512689.
doi:10.1001/jamanetworkopen.2025.12689

eTable 1. *ICD* and *CPT* codes used for patient selection
eTable 2. mFI-5 scoring criteria
eTable 3. RAI Scoring criteria
eTable 4. Weighting of age variable by presence of cancer for RAI
eFigure. CONSORT Flow Diagram
eTable 5. Crosstabulation of mFI-5 and RAI
eTable 6. Discriminatory accuracy of the RAI and mFI-5 for primary outcome and secondary outcomes
eTable 7. Multivariable analysis controlling for age, sex, race, operative time, and transfer status, for prediction of 30-day outcomes by the RAI and mFI-5

This supplemental material has been provided by the authors to give readers additional information about their work.

© 2025 Gupta N et al. *JAMA Network Open*.

eTable 1. *ICD* and *CPT* codes used for patient selection

Code Type	Code	Description
ICD-9	820.x	Fracture of neck of femur
ICD-10	S72.0xxx	Fracture of head and neck of femur
	S72.1xxx	Pertrochanteric fracture
CPT	27236	Open treatment (fixation or prosthetic)
	27244	Plate/screw fixation (sliding compression screw)
	27245	Intramedullary implant (cephalomedullary nail)
	27130	Total Hip Arthroplasty
	27125	Hemiarthroplasty

© 2025 Gupta N et al. *JAMA Network Open*.

eTable 2. mFI-5 scoring criteria

mFI-5 (Component)	Score (Maximum = 5)
Non Independent Functional Status (Partial and Complete)	1
Diabetes mellitus with oral agents or insulin	1
COPD	1
Hypertension Requiring Medication	1
Congestive Heart Failure	1

© 2025 Gupta N et al. *JAMA Network Open*.

eTable 3. RAI Scoring criteria

**RAI (Component)**	**Score**
Sex	+3 if male
Age	+Score weighted by cancer (Supplementary Table 4)
Cancer diagnosis (excluding skin cancer, except melanoma)	Relevant through age variable
Unintentional weight loss >4.5kg over 3 months	+4
Renal failure (and/or dialysis)	+8
CHF	+5
Poor appetite	+4
Shortness of breath at rest	+3
Residency other than independent living (transferred from nonhome or intermediate care unit)	+1
Cognitive deterioration (over past 3 months)	N/A
Activities of daily living	+14 = totally dependent<br>+7 = partially dependent<br>+0 = independent

© 2025 Gupta N et al. *JAMA Network Open*.

eTable 4. Weighting of age variable by presence of cancer for RAI

Age	Score with cancer	Score without cancer
≤ 19	28	0
20-24	29	1
25-29	29	4
30-34	30	6
35-39	30	8
40-44	31	10
45-49	31	12
50-54	32	14
55-59	32	16
60-64	33	18
65-69	34	20
70-74	34	22
75-79	35	24
80-84	35	26
85-89	36	28

© 2025 Gupta N et al. *JAMA Network Open*.

eFigure. CONSORT Flow Diagram

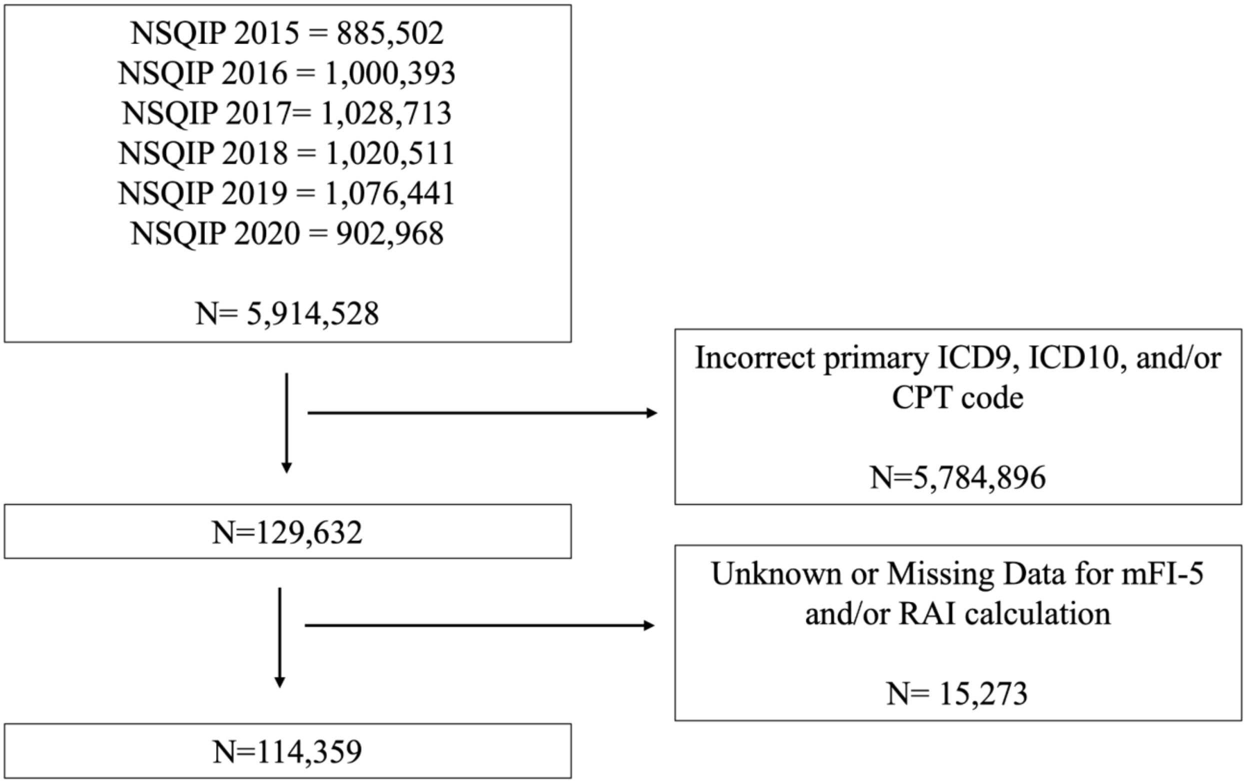

© 2025 Gupta N et al. *JAMA Network Open*.

eTable 5. Crosstabulation of mFI-5 and RAI

	RAI Frailty Tiers<br>No. (%)				
Percent Composition of mFI-5 Tier		**Robust**	**Normal**	**Frail**	**Very Frail**
	**Nonfrail**	8,252 (32.3)	6,626 (24.3)	6,395 (21.5)	1,838 (5.8)
	**Prefrail**	10,957 (42.8)	14,480 (53.2)	15,864 (53.4)	9,770 (30.6)
	**Frail**	5,841 (22.8)	5,522 (20.3)	5,913 (19.9)	14,154 (44.4)
	**Severely Frail**	522 (2.0)	589 (2.2)	1,515 (5.1)	6,121 (19.2)

© 2025 Gupta N et al. *JAMA Network Open*.

eTable 6. Discriminatory accuracy of the RAI and mFI-5 for primary outcome and secondary outcomes

Odds Ratio (95% Confidence Interval)			
	**mFI-5**	**RAI**	**P-Value**
**Primary Endpoints**			
Mortality	0.61 (0.60-0.62)	0.73 (0.72-0.73)	<0.001
**Secondary Endpoints**			
eLOS	0.55 (0.55-0.56)	0.57 (0.57-0.57)	<0.001
NHD	0.51 (0.51-0.52)	0.49 (0.48-0.49)	<0.001
Readmission	0.57 (0.56-0.57)	0.56 (0.55-0.56)	0.007
Reoperation	0.53 (0.51-0.54)	0.50 (0.49-0.51)	<0.001
Complications	0.58 (0.57-0.59)	0.58 (0.57-0.58)	0.413

© 2025 Gupta N et al. *JAMA Network Open*.

eTable 7. Multivariable analysis controlling for age, sex, race, operative time, and transfer status, for prediction of 30-day outcomes by the RAI and mFI-5

	Odds Ratio (95% Confidence Interval)					
	mFI-5			RAI		
	Prefrail<br>1	Frail<br>2	Severely Frail<br>≥ 3	Normal<br>26-28	Frail<br>29-32	Very Frail<br>≥ 33
**Mortality**	1.35 (1.24-1.47)	2.11 (1.94-2.30)	3.53 (3.20-3.90)	1.55 (1.35-1.79)	2.97 (2.59-3.42)	6.17 (5.38-7.08)
**eLOS**	1.23 (1.19-1.28)	1.61 (1.55-1.68)	2.32 (2.19-2.45)	1.43 (1.36-1.50)	1.70 (1.61-1.79)	2.18 (2.06-2.31)
**NHD**	1.16 (1.12-1.20)	1.17 (1.12-1.21)	1.11 (1.05-1.17)	1.42 (1.35-1.49)	1.44 (1.36-1.52)	0.93 (0.88-0.99)
**Major Complications**	1.39 (1.29-1.50)	1.83 (1.69-1.98)	2.91 (2.65-3.19)	1.48 (1.35-1.63)	1.87 (1.69-2.07)	2.27 (2.04-2.51)
**Readmission**	1.34 (1.26-1.43)	1.70 (1.59-1.82)	2.47 (2.27-2.69)	1.43 (1.32-1.55)	1.77 (1.62-1.93)	2.17 (1.99-2.37)
**Reoperation**	1.17 (1.05-1.30)	1.25 (1.11-1.40)	1.50 (1.28-1.74)	1.33 (1.17-1.52)	1.33 (1.14-1.54)	1.44 (1.27-1.68)

© 2025 Gupta N et al. *JAMA Network Open*.
