# Supplementary material for: Risk Analysis Index for Estimation of 30-Day Postoperative Mortality in Hip Fractures
Source: JAMA Netw Open. 2025 May 29;8(5):e2512689. doi: 10.1001/jamanetworkopen.2025.12689 (PMC12123473; doi:10.1001/jamanetworkopen.2025.12689)
Supplement: Supplement 2. — Data Sharing Statement [file jamanetwopen-e2512689-s002.pdf]

Data Sharing Statement

Gupta. Risk Analysis Index for Estimation of 30-Day Postoperative Mortality in Hip Fractures. *JAMA Netw Open*. Published May 29, 2025. doi:10.1001/jamanetworkopen.2025.12689

Data
Data available: Yes
Data types: Deidentified participant data
How to access data: All patient data, as well as diagnostic and procedural billing codes, are available on reasonable request of the corresponding author following completion of necessary onboarding procedures specified by the American College of Surgeons National Surgical Quality Improvement Program.
When available: With publication

Supporting Documents
Document types: None

Additional Information
Who can access the data: For any purpose as allowed by the American College of Surgeons National Surgical Quality Improvement Program.
Types of analyses: For any purpose as allowed by the American College of Surgeons National Surgical Quality Improvement Program.
Mechanisms of data availability: After completion of necessary onboarding procedures specified by the American College of Surgeons National Surgical Quality Improvement Program.
